# The Association of Pseudohypoparathyroidism Type Ia with Chiari Malformation Type I: A Coincidence or a Common Link?

**DOI:** 10.1155/2016/7645938

**Published:** 2016-09-14

**Authors:** Paria Kashani, Madan Roy, Linda Gillis, Olufemi Ajani, M. Constantine Samaan

**Affiliations:** ^1^Department of Pediatrics, McMaster University, Hamilton, ON, Canada; ^2^Division of Neurosurgery, McMaster Children's Hospital, Hamilton, ON, Canada; ^3^Division of Pediatric Endocrinology, McMaster Children's Hospital, Hamilton, ON, Canada

## Abstract

A 19-month-old boy was referred for progressive weight gain. His past medical history included congenital hypothyroidism and developmental delay. Physical examination revealed characteristics of Albright Hereditary Osteodystrophy, macrocephaly, and calcinosis cutis. He had hypocalcemia, hyperphosphatemia, and elevated Parathyroid Hormone levels. Genetic testing revealed a known mutation of GNAS gene, confirming the diagnosis of Pseudohypoparathyroidism Type Ia (PHP-Ia) (c.34C>T (p.G1n12X)). He had a normal brain MRI at three months, but developmental delay prompted a repeat MRI that revealed Chiari Malformation Type I (CM-I) with hydrocephalus requiring neurosurgical intervention. This was followed by improvement in attaining developmental milestones. Recently, he was diagnosed with growth hormone deficiency. This case suggests the potential association of CM-I with PHP-Ia. Larger studies are needed to assess whether CM-I with hydrocephalus are common associations with PHP-Ia and to define potential genetic links between these conditions. We propose a low threshold in performing brain MRI on PHP-1a patients, especially those with persistent developmental delay to rule out CM-I. Early intervention may improve neurodevelopmental outcomes and prevent neurosurgical emergencies.

## 1. Introduction

Pseudohypoparathyroidism Type Ia (PHP-Ia) is the most common form of pseudohypoparathyroidism. It is characterized by end organ resistance to several hormones. PHP-Ia is an imprinting defect, with heterozygous loss of function mutation of Guanine nucleotide binding protein, alpha subunit 1 (GNAS1) gene on the maternal allele. This defect results in singular expression of paternally inherited GNAS1 allele [1]. The mutation reduces the G-protein alpha- (Gs*α*-) adenylate cyclase complex signaling and results in reduction of cyclic AMP production. cAMP serves as a second messenger in G-protein coupled receptor signaling for several hormones [2].

The most common hormonal resistance noted in PHP-Ia is Thyroid Stimulating Hormone (TSH) and Parathyroid Hormone (PTH). PHP-Ia can also be associated with a characteristic phenotype termed Albright Hereditary Osteodystrophy (AHO) [3], with round facies, short stature, and brachydactyly. Some of these patients also have growth hormone deficiency [4].

Intellectual disability is present in 45–75% of patients with PHP-Ia, and it has been proposed that Gs is imprinted in the brain [5, 6]. Obesity is a common feature in PHP-Ia and is associated with reduced resting energy expenditure [7] and reduced lipolysis, which is due to resistance to epinephrine [8].

Chiari type I malformation (CM-I) is characterized by elongation of cerebellar tonsils into the cervical canal via foramen magnum, which can compress the herniated tissue [9]. Symptomatic CM-I is usually associated with cervical syringomyelia and hydrocephalus [10]. Patients with CM-I can present with a spectrum of symptoms including sleep apnea [11], irritability, failure to thrive, and developmental delay [12].

In this report, we present a case of PHP-Ia associated with CM-I in a patient referred for management of obesity and discuss potential links. Consent was obtained from the patient's mother for the publication of this report.

## 2. Case Presentation

A 19-month-old boy was referred to a tertiary pediatric center for management of obesity.

He was born to a 36-year-old healthy mother who had two other healthy children from the same partner. There was no history of gestational diabetes mellitus or preeclampsia. His mother did not smoke, consume alcohol, or use drugs during pregnancy. The patient was born at 39 weeks of gestation by elective Cesarean section, due to the previous history of sections with no complications. His birth weight was 3.03 kg (25–50th percentile) and birth length was 47 cm (10–25th percentile) with no history of neonatal jaundice, hypotonia, tube feeding, or hypoglycemia. Family history was significant for hypothyroidism in maternal grandmother. The family did not report developmental delay in other family members.

His newborn screening test for hypothyroidism (TSH) was normal. At three weeks of age, he developed* Klebsiella* sepsis and responded to intravenous antibiotic therapy.

At three months of age, he had documented excessive weight gain (approximately 1.2 Kg/month between 3 to 5 months of age) while his food intake was reported to be within the normal range for age.

He had hypothyroidism diagnosed at five months of age, with elevated TSH and low free T4 and was on thyroid hormone supplements, but his weight gain continued despite this. Physical examination at five months revealed no goiter, coarse features, hypotonia, dry skin, macroglossia, or umbilical hernia. At seven months of age, he was noted to be hypotonic and exhibited fine and gross motor delay.

On presentation, his weight was 20.1 kg (above 95th percentile for sex and age) and his length was 83.5 cm (50–75th percentile), with BMI of 28.8 kg/m^2^ (above 95th percentile for sex and age).

Physical examination revealed dysmorphic features including macrocephaly, low set ears, frontal bossing, rounded facies, and depressed nasal bridge with upturned nose. He also had multiple white papulonodular lesions on the right hand and trunk, consistent with calcinosis cutis. Hand examination revealed no evidence of brachydactyly. Neurological exam revealed gross motor delay, as he could not be able to stand without support and was not walking. In addition, his fine motor and expressive language were delayed with only a few words spoken.

Table 1 includes initial blood tests done to complete his evaluation. He had hypocalcemia, hyperphosphatemia, and elevated PTH with normal vitamin D levels.

X-ray of the left hand and wrist for bone age revealed a bone age of six years at a chronological age of 2.5 years, which is advanced. The length of 4th and 5th metacarpal bones was normal.

Due to the clinical suspicion of syndromic obesity, genetic testing was done and revealed heterozygosity for a known mutation of GNAS1 gene (c.34C>T (p.G1n12X)), confirming the diagnosis of Pseudohypoparathyroidism Type Ia (PHP-Ia).

**Figure 1:**
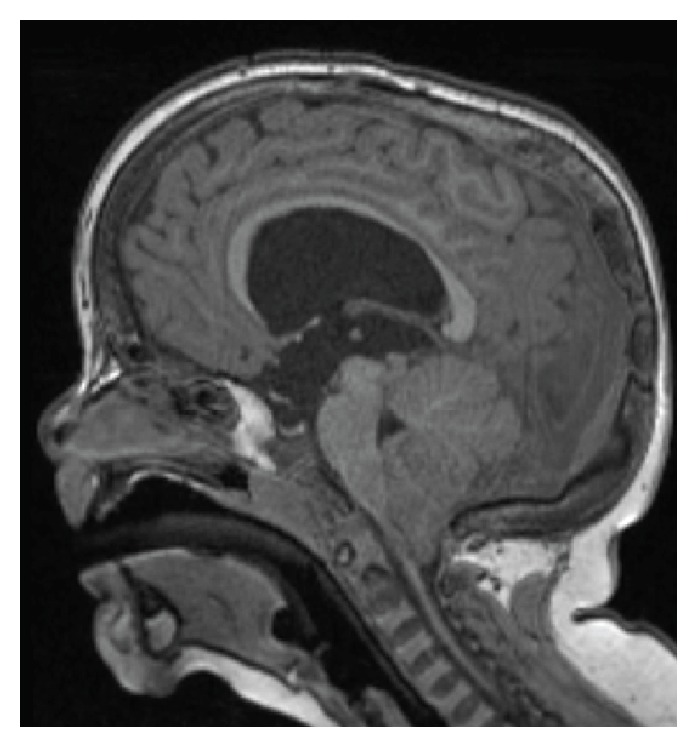
Brain MRI showing hydrocephalus and CM-I.

On subsequent follow-ups, persistent developmental delay prompted brain and spine MRI at the age of 34 months to rule out underlying neurological causes of developmental delay and to examine for cerebral calcifications. This MRI demonstrated Chiari Malformation Type I (CM-I) with hydrocephalus (Figure 1). The patient required neurosurgical intervention with endoscopic ventriculostomy for intracranial pressure relief with a satisfactory outcome.

For weight management, recommendations for caloric intake and physical activity were implemented. Alphacalcidol (0.015 *μ*g/kg/day) that was then switched to calcitriol at the same dose, calcium carbonate (100 mg/kg/day start-up dose), and vitamin D supplementation (800 IU/day) were initiated.

The BMI improved within six months of lifestyle intervention to 21.6 kg/m^2^. Calcium homeostasis was normalized with pharmacotherapy. Diet was strictly monitored for calcium and vitamin D intake and pharmacotherapy altered based on intake.

Attainment of developmental milestones improved significantly after ventriculostomy. The patient received occupational therapy, physiotherapy, and speech therapy to help with the different areas of development including fine motor and gross motor development and speech.

As he had low IGF-1, he was suspected of having growth hormone deficiency, and this diagnosis was confirmed at 5.3 years of age based on Arginine and Clonidine stimulation tests.

## 3. Discussion

In this paper, we report a case of PHP Type Ia combined with CM-I. PHP-Ia is the most common form of pseudohypoparathyroidism, and this patient is younger than the one case report we are aware of that has reported an association for PHP-Ia and CM-I [13]. He is currently having ear, nose, and throat evaluations and sleep study before initiation of growth hormone therapy.

While the causes of CM-I in PHP-Ia are still unknown, there is some evidence that growth hormone deficiency leads to abnormal development of the posterior fossa bone growth [14]. When the posterior fossa volume and morphology in children with growth hormone deficiency with and without CM-I were compared, the volumes of the posterior fossa were similar, yet the morphology of the posterior fossa in both groups was different compared to growth hormone sufficient controls, with measurable underdevelopment of parts of the bony structures of the posterior fossa [14].

Further support for the role of growth hormone in CM-I stems from that fact that growth hormone insensitivity in CM-I reproduces a similar phenotype of CM-I to that of growth hormone deficient children [15]. It is already established that, in PHP-Ia, there is insensitivity to the hypothalamic growth hormone-releasing hormone (GHRH) [16]. Growth hormone and GHRH insensitivity may impact occipital bone development in PHP-Ia and leads to CM-I, and this requires further study. Our patient was recently diagnosed with growth hormone deficiency, which may be related to his findings.

Another potential explanation for the relationship between PHP-Ia and CM-I may be due to genetic associations. This is supported by evidence of higher prevalence of CM-I in twins and family members of those with CM-I [17, 18] and the association of CM-I with certain genetic syndromes [19]. Whether there is a common genetic link between PHP-Ia and CM-I remains unknown, and combined imaging and genetic association studies are needed to clarify this possibility.

Our patient had a normal MRI brain early in life and an abnormal MRI at the time of diagnosis of CM-I. This suggests that, in this case, CM-I is a progressive anomaly that may have developed due to hydrocephalus, and this is compounded by reduced growth hormone action on the posterior fossa growth, which may contribute to developmental delay. Longitudinal studies are needed to determine if CM-I contributes to the neurodevelopmental delay in PHP-Ia.

Clinicians should be mindful of the association of PHP-Ia and CM-I. It is important to consider brain MRI in PHP-Ia patients, particularly those with abnormal neurological examination or developmental delay. While developmental delay may be a feature of PHP-Ia, detecting CM-I with hydrocephalus may help to maximize neurodevelopmental outcomes and prevent neurosurgical emergencies. Further research into the genetic links between these two conditions is warranted.

## Figures and Tables

**Table 1:** Initial laboratory investigations.

Laboratory test	Measured value	Reference intervals
PTH	53.5 pmol/L (H)	1.5–7.2 pmol/L
Total calcium	1.92 mmol/L (L)	2.30–2.62 mmol/L
Inorganic phosphate	2.72 mmol/L (H)	1.39–2.20 mmol/L
Magnesium	0.89 mmol/L	0.62–0.95 mmol/L
Albumin	42 g/L	35–45 g/L
Alkaline phosphatase	375 U/L (H)	156–369 U/L
25-Hydroxyvitamin D	53.0 nmol/L	50–250 nmol/L
1,25-Dihydroxyvitamin D	60 pmol/L	39–193 pmol/L
IGF-1	36 *μ*g/L (L)	63–279 *μ*g/L
TSH	34 mU/L (H)	1.4–8.8
Free T4	6.8 pmol/L (L)	13.9–26.1
